# Eosinophilic Upper Airway Inflammation in a Murine Model Using an Adoptive Transfer System Induces Hyposmia and Epithelial Layer Injury with Convex Lesions

**DOI:** 10.3390/medsci7020022

**Published:** 2019-02-05

**Authors:** Akira Kanda, Kenji Kondo, Naoki Hosaka, Yoshiki Kobayashi, Dan Van Bui, Yasutaka Yun, Kensuke Suzuki, Shunsuke Sawada, Mikiya Asako, Akihiko Nakamura, Koichi Tomoda, Yoshiko Sakata, Koji Tsuta, David Dombrowicz, Hideyuki Kawauchi, Shigeharu Fujieda, Hiroshi Iwai

**Affiliations:** 1 Department of Otolaryngology, Head and Neck Surgery, Kansai Medical University, Hirakata 573-1010, Japan; kobayosh@hirakata.kmu.ac.jp (Y.K.); buivanda@hirakata.kmu.ac.jp (D.V.B.); yunys@hirakata.kmu.ac.jp (Y.Y.); suzukken@hirakata.kmu.ac.jp (K.S.); sawadash@hirakata.kmu.ac.jp (S.S.); asako@hirakata.kmu.ac.jp (M.A.); tomodak@hirakata.kmu.ac.jp (K.T.); iwai@hirakata.kmu.ac.jp (H.I.); 2 Allergy Center, Kansai Medical University, Hirakata 573-1010, Japan; 3 Department of Otolaryngology and Head and Neck Surgery, Graduate School of Medicine, the University of Tokyo Hospital, Tokyo, 113-8655, Japan; kondok-tky@umin.ac.jp; 4 Department of Pathology, Fuchu Hospital, Izumi 594-0076, Japan; hosakan@hirakata.kmu.ac.jp; 5 Nakamura ENT Clinic, Sakai 591-8025, Japan; nakamura-ent@paw.hi-ho.ne.jp; 6 Central Research Laboratory, Kansai Medical University, Hirakata 573-1010, Japan; sakatayo@hirakata.kmu.ac.jp; 7 Department of Pathology, Kansai Medical University, Hirakata 573-1010, Japan; tsutakoj@hirakata.kmu.ac.jp; 8 EGID, Inserm, CHU Lille, Institut Pasteur de Lille, U1011, University of Lille, 59019 Lille, France; david.dombrowicz@pasteur-lille.fr; 9 Department of Otorhinolaryngology, Shimane University Faculty of Medicine, Izumo 693-0021, Japan; kawauchi@med.shimane-u.ac.jp; 10 Department of Otorhinolaryngology Head & Neck Surgery, University of Fukui, Fukui 910-1193, Japan; sfujieda@g.u-fukui.ac.jp

**Keywords:** allergic rhinitis, chronic rhino sinusitis, nasal poly, eosinophil, hyposmia

## Abstract

Background: Chronic rhinosinusitis with nasal polyps (CRSwNP) is a refractory upper airway disease, accompanied mainly by eosinophilia and/or asthma. In addition, the disease correlates with a high rate of hyposmia, following a marked infiltration of eosinophils into the inflamed site, the paranasal sinus. Although eosinophils are known to contribute to the development of hyposmia and CRSwNP pathology, the underlying mechanisms remain unclear. This study aimed to investigate whether eosinophilic upper airway inflammation induces hyposmia and CRSwNP in a murine model using an adoptive transfer system. Methods: To induce eosinophilic rhinosinusitis, splenocytes, including a high proportion (over 50%) of activated eosinophils (SPLhEos), were collected from interleukin-5 transgenic mice following double intraperitoneal injections of antigens, such as ovalbumin, house dust mite, or fungus. Activated SPLhEos with corresponding antigens were then transferred into the nasal cavity of recipient mice, which were sensitized and challenged by the corresponding antigen four times per week. Olfactory function, histopathological, and computed tomography (CT) analyses were performed 2 days after the final transfer of eosinophils. Results: Hyposmia was induced significantly in mice that received SPLhEos transfer compared with healthy and allergic mice, but it did not promote morphological alteration of the paranasal sinus. Pathological analysis revealed that epithelial layer injury and metaplasia similar to polyps, with prominent eosinophil infiltration, was induced in recipient tissue. However, there was no nasal polyp development with interstitial edema that was similar to those recognized in human chronic rhinosinusitis. Conclusions: This study supports the previously unsuspected contribution of eosinophils to CRS development in the murine model and suggests that murine-activated eosinophilic splenocytes contribute to the development of hyposmia due to more mucosal inflammation than physical airway obstruction and epithelial layer injury with convex lesions.

## 1. Introduction

Eosinophils play a critical role in Th2 (T-helper cell type 2)-associated pathology of diseases, such as allergic rhinitis (AR), Th2-skewed chronic rhinosinusitis (CRS), and asthma [1,2,3]. Eosinophils exert their effect through cytotoxic mediators, comprising granules (eosinophil peroxidase (EPO), major basic protein (MBP), eosinophil cationic protein (ECP), and eosinophil-derived neurotoxin (EDN)), cytokines (Interleukin (IL)-2, IL-4, IL-5, IL-6, IL-10, IL-13, IL-16, IL-18, and transforming growth factor (TGF)-β), chemokines (regulated on activation, normal T cell expressed and secreted (RANTES) and eotaxin), and lipid mediators (platelet-activating factor and leukotriene C4) [1,2,3]. Eosinophil-derived cytotoxic mediators cause damage to the epithelial layer, airway mucosa, and nerves, resulting subsequently in airway hyperresponsiveness (AHR). Furthermore, profibrotic cytokines and fibrogenic mediators, such as IL-11, IL-17, IL-17E (also known as IL-25), TGF-α, TGFβ1, and matrix metalloproteinase-9, are involved in airway remodeling in asthma [4] and polyp formation in CRS with nasal polyps (CRSwNP) [5]. However, the development of CRSwNP pathogenesis is still poorly understood.

Chronic rhinosinusitis is currently grouped into clinical phenotypes of CRSwNP and CRS without nasal polyps (CRSsNP). Patients with CRSwNP possess a high risk of recurrent sinonasal polyps following endoscopic sinus surgery (ESS) [6], and CRSwNP is closely associated with asthma and characterized by eosinophilia. On the other hand, CRSsNP is generally accompanied by bacterial infection and/or the presence of neutrophils [6]. Regarding mechanisms of CRSwNP development, Bachert et al. previously reported that both orchestration by Th2 cytokines and amplification by *Staphylococcus aureus* enterotoxin B (SEB), a *S. aureus* superantigen, are required for the formation of nasal polyps with eosinophilia [7]. Furthermore, in a recent study of inflammatory endotypes and phenotypes of CRS, CRSwNP, and CRSsNP, based on cluster analysis of biomarkers, Tomassen et al. demonstrated that high expression of IL-5 and the presence of *S. aureus* enterotoxin-specific IgE (SE-IgE) were both observed in patients with CRSwNP, but not in those with CRSsNP [8].

Given that the affected tissue in patients with CRSwNP is frequently infiltrated by large numbers of eosinophils, the name “eosinophilic rhinosinusitis” (ECRS) has been proposed as a new clinically diagnosed phenotype of CRSwNP [9,10]. ECRS is characterized by blood eosinophilia, ethmoid sinus disease detected by computed tomography (CT), bronchial asthma, and aspirin and nonsteroidal anti-inflammatory drug intolerance in CRSwNP [9,10]. Regarding clinical symptoms, the development of hyposmia or anosmia in particular commonly precedes other symptoms, such as nasal obstruction, and is significantly exacerbated in patients with ECRS compared with non-ECRS in CRSwNP [9,10]. Similarly, Klimek et al. previously reported that olfactory dysfunction following specific antigen provocation in patients with grass pollen sensitivity is correlated more closely with the level of inflammatory eosinophil-derived cytotoxic mediators, such as ECP, in nasal secretions than with nasal flow volume measured by active anterior rhinomanometry, suggesting a relationship between olfactory dysfunction and nasal eosinophilic inflammation [11]. Thus, these data indicate that eosinophils directly and/or indirectly cause olfactory damage in inflamed sites. However, no reports exist as to whether eosinophils are capable of directly inducing olfactory dysfunction in ECRS as well as AR.

Understanding the mechanisms behind nasal polyp formation and better informing drug discovery research for ECRS in CRSwNP require not only cluster analyses of human samples but also the development of an animal model of CRSwNP. Concerning the development of CRSwNP in murine models, Kim et al. reported that nasal polypoid lesions could be induced in an AR murine model treated with ovalbumin (OVA) plus SEB [12]. However, studies using this animal disease model have been reported by this one group [12,13,14].

To assess the essential role of eosinophils in vivo, our group previously reported an eosinophil-derived airway inflammation model via eosinophil transfer into the lower airway of recipient mice through intratracheal administration [15]. In this study, we examined whether splenocytes (containing a large number of eosinophils) transferred into a recipient’s nasal cavity can induce CRSwNP with hyposmia.

## 2. Methods

### 2.1. Mice

The following mouse strains were used: BALB/c and IL-5 transgenic (Tg) mice (BALB/c background), obtained from Shimizu Laboratory (Kyoto, Japan) and Dr. D. Dombrowicz (Institut Pasteur de Lille, Lille, France), respectively. All mice were housed at 21–23 °C with 40–60% humidity in animal facilities with a 12 h light/dark cycle and were provided food and water ad libitum. All animal experiments were performed using protocols approved by the Kansai Medical University Animal Ethics Committee (18-082).

### 2.2. Preparation of Splenocytes including a High Number of Activated Eosinophils

**Figure 1:**
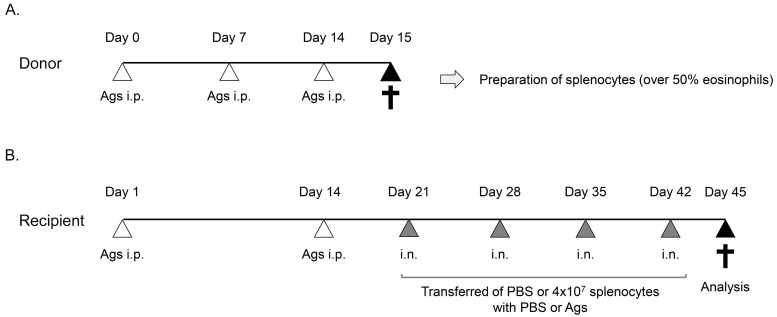
Experimental design of the adoptive transfer system. (**A**). Preparation of activated splenocytes from the donor. Donor IL-5 Tg mice were sensitized with three intraperitoneal (i.p.) injections of antigens (Ags): ovalbumin (OVA), *Dermatophagoides farinae* (Der f), or *Aspergillus*. Splenocytes including a high number of eosinophils (SPLhEos) were collected from spleens of IL-5 Tg mice 24 h after the final injection. (**B**) Protocol for adoptive transfer into recipient mice. After recipient mice had been sensitized with (i.p.) injections of corresponding Ags for the donor, 4 × 10^7^ splenocytes were transferred into the nasal cavity of recipient mice via intranasal (i.n.) injections. “Control animals” indicates transfer of PBS or SPLhEos with phosphate buffered saline (PBS). Simultaneously, PBS or corresponding Ags were also administrated alongside SPLhEos via i.n. injections.

To collect activated splenocytes including high proportions of eosinophils (SPLhEos), donor mice (IL-5 Tg) were sensitized with three intraperitoneal injections of PBS or antigen: 50 µg OVA (grade V; Sigma, St. Louis, Missouri, USA), 10 µg *Dermatophagoides farinae* (Der f) house dust mite (HDM) allergen (Institute of Tokyo Environmental Allergy, Tokyo, Japan), or 50 µg *Aspergillus* (Institute of Tokyo Environmental Allergy, Tokyo, Japan) in 2 mg of aluminum hydroxide (Alum) (Thermo Fisher Scientific, Waltham, Massachusetts, USA) on Days 0, 7, and 14 (Figure 1A). Donor mice were sacrificed at Day 15, and splenocytes were prepared. Cells were counted in a hemocytometer and using Diff Quik (Dade Behring AG, Dudingen, Switzerland) following cytospin (Thermo Shandon, Pittsburgh, PA, USA). The eosinophil percentage among splenocytes was greater than 50%. The character of splenocytes from IL-5 Tg mouse as determined by flow cytometric analysis is shown in Figure S1.

### 2.3. Adoptive Transfer System

To induce activated ECRS in a murine model, an eosinophil adoptive transfer system was designed, as shown in Figure 1B [15,16]. Recipient mice were sensitized with PBS or corresponding antigens on Days 0 and 14. Following splenocyte preparation, 2 × 10^6^ activated SPLhEos in 30 µL phosphate-buffered saline (PBS) alone, PBS with 0.5% OVA, 1 µg Der f, or 10 µg *Aspergillus* (or the same allergens alone in PBS, or PBS alone) were transferred into the nasal cavity of recipients sensitized with PBS or corresponding antigens by intranasal administration into both nostrils, once per week (4 times at each instance) for 4 weeks, on Days 21, 28, 35, and 42. Administration was performed under systemic anesthesia with 0.5 mg/kg medetomidine (Domitor; Pfizer, New York, NY, USA) and 50 mg/kg ketamine (Ketalar; Daiichi-Sankyo, Tokyo, Japan). In HDM- and *Aspergillus*-sensitized mice, 1 mg/µL SEB (Sigma) or 2 µM DNA containing unmethylated CpG motifs (CpGDNA: ODN2395; type B; Cosmo Bio, Tokyo, Japan) was also administered along with SPLhEos. Recipient mice were then evaluated via a functional assay (buried food test), histological analysis, and CT analysis at 48 h post the final SPLhEos transfer.

### 2.4. Buried Food Test

Buried food tests to evaluate the olfactory function of mice were performed 48 h after the final SPLhEos according to Yang et al.’s method, with some modifications [17]. Food deprivation was initiated 24 h prior to the test by removing pellets from the food hopper of the home cage. During the test, a clean cage was prepared (44 cm L × 29.2 cm W × 20 cm H) containing a 3 cm depth of clean bedding. The subject mouse was transferred to the test cage, allowed to acclimatize for 5 min, and then returned to the original cage. After the mouse had been returned to the home cage, one piece of cookie (Tabekko Dobutsu Biscuits, Ginbisu, Tokyo, Japan) was buried in a random corner, approximately 1 cm beneath the surface of the test cage. The subject mouse was then transferred to the middle of the test cage and latency was measured between the time point of transfer and the subject mouse finding the buried food. If the subject had failed to find the buried food after 5 min had elapsed, the test was stopped and 300 s was recorded as the latency score.

### 2.5. Histological Analysis

To prepare paraffin sections 48 h after the final transfer of SPLhEos, recipient mice were fixed through cardiac perfusion with 10% neutral buffered formalin (Muto Kagaku, Tokyo, Japan) under deep anesthesia and decapitated. Trimmed heads, including nasal cavity, were then locally irrigated using the same fixative at room temperature for 1 week, followed by decalcification in 10% ethylenediaminetetraacetic acid (pH 7.0) at room temperature for 2 weeks. Following decalcification, coronal sections (4 μm thick) on the level of the anterior end of the olfactory bulb were mounted on Matsunami-adhesive-silane (MAS)-coated slides (Matsunami Glass, Osaka, Japan). For human samples, the use of nasal polyps from patients with CRSwNP was approved by the local ethics committee of University of Tokyo Hospital (12009).

To prepare frozen sections, following decapitation, trimmed heads were immediately frozen in isopentane (nacalai tesque, Kyoto, Japan) cooled in liquid nitrogen, then freeze-embedded with super cryoembedding medium (SCEM, Leica Microsystems, Land Hessen, Germany) in coolant. Fresh frozen samples were sectioned using a film method without fixation or decalcification (the “unfixed and undecalcified method”), known as the Kawamoto method [18]. Fresh-frozen sections (4 µm thick) were mounted on Cryofilm (Leica Microsystems).

Sections were stained with hematoxylin and eosin (H&E) for morphological analysis or Sirius red staining for visualization of eosinophils. Histological analyses were assessed by two different pathologists.

### 2.6. Computed tomography Analysis

Computed tomography scans were performed immediately on mice sacrificed 48 h after the last transfer, using a Siemens Inveon Micro-CT (Siemens, Bayern, Germany). Images were calibrated for Hounsfield unit scaling using a water-filled phantom on each experiment day. The scanner settings were as follows: tube voltage of 70 kVp and current of 500 μA over 360 continuous projections with an exposure time of 1000 ms per projection. Cross-sectional images were reconstructed using Inveon Viewer Quick Launch (Siemens) and converted to the Digital Imaging and Communications in Medicine (DICOM) format using PMOD software (PMOD Technologies LLC, Zurich, Switzerland).

### 2.7. Statistical Analysis

Data are presented as means ± standard errors of the mean (SEMs). Statistical significance was determined using the Mann–Whitney U test in the buried food test. The threshold of significance was set at *p* < 0.05 for all tests.

A Supplementary Materials section can be found in the online repository for this article.

## 3. Results

### 3.1. Hyposmia on Transfer of SPLhEos in Th2-Skewed Response

**Figure 2:**
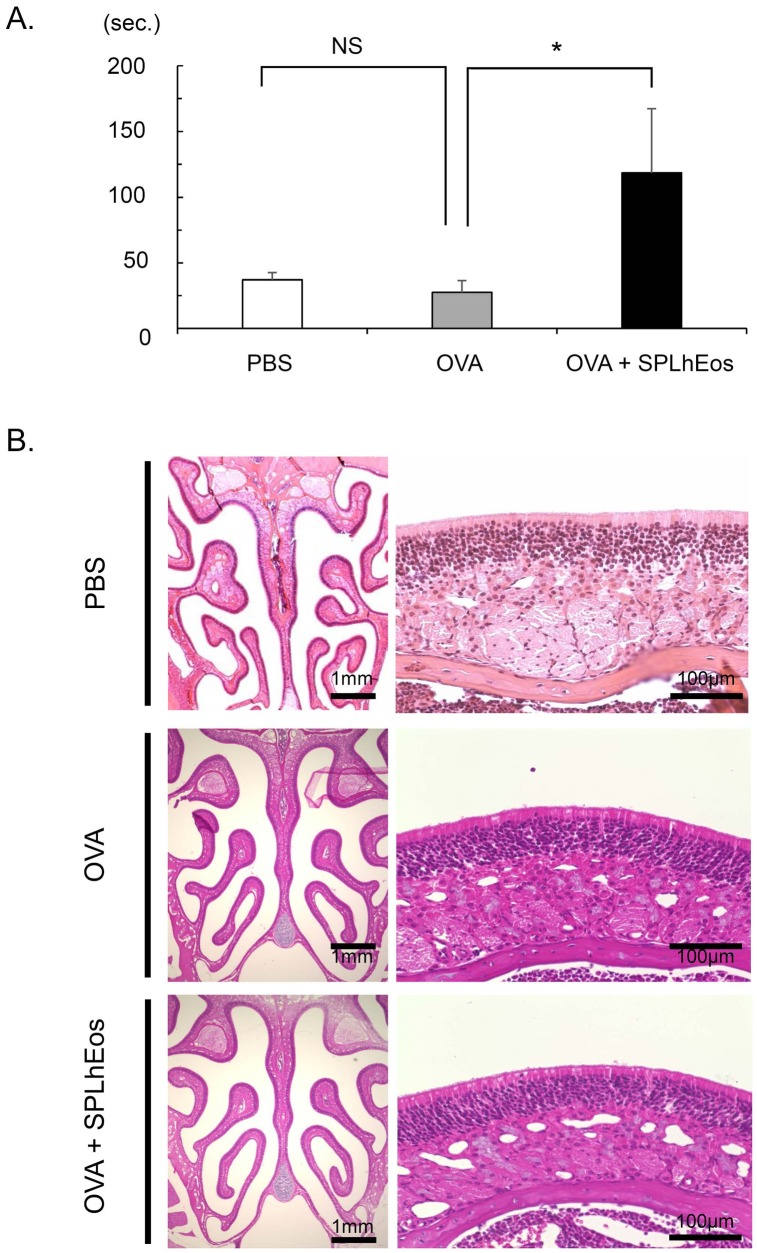
Buried food test (**A**) and histological analysis (**B**) following the transfer of SPLhEos in Th2-skewed response. (**A**.) Open, gray, and black bars indicate the transfer of PBS alone, OVA alone, and OVA + SPLhEos (4 × 10^7^ cells) in the adoptive transfer system, respectively. Data are expressed as means ± SEM of *n* = 5 mice per group. * Statistically significant difference from control mice (*p* < 0.05). (**B**) Hematoxylin and eosin (H&E) staining of histological sections.

To investigate the functional role of Th-2 skewed SPLhEos in vivo, an adoptive transfer system was performed with Th2 polarization. SPLhEos activated by OVA were transferred into OVA-sensitized recipients; the buried food test was then performed to assess olfactory dysfunction, followed by histological analysis. As shown in Figure 2A, the olfactory dysfunction assay revealed that hyposmia was significantly induced on adoptive transfer of SPLhEos with OVA in recipient mice (118 ± 48.8 s) compared with OVA alone (27.6 ± 8.9 s) (*p* = 0.047). However, there were no clear differences in morphological change of the paranasal sinus (in paraffin sections) between groups, or in the development of nasal polypoid lesions (Figure 2B).

### 3.2. Epithelia Injury on Transfer of SPLhEos in Adoptive and Innate Responses

**Figure 3:**
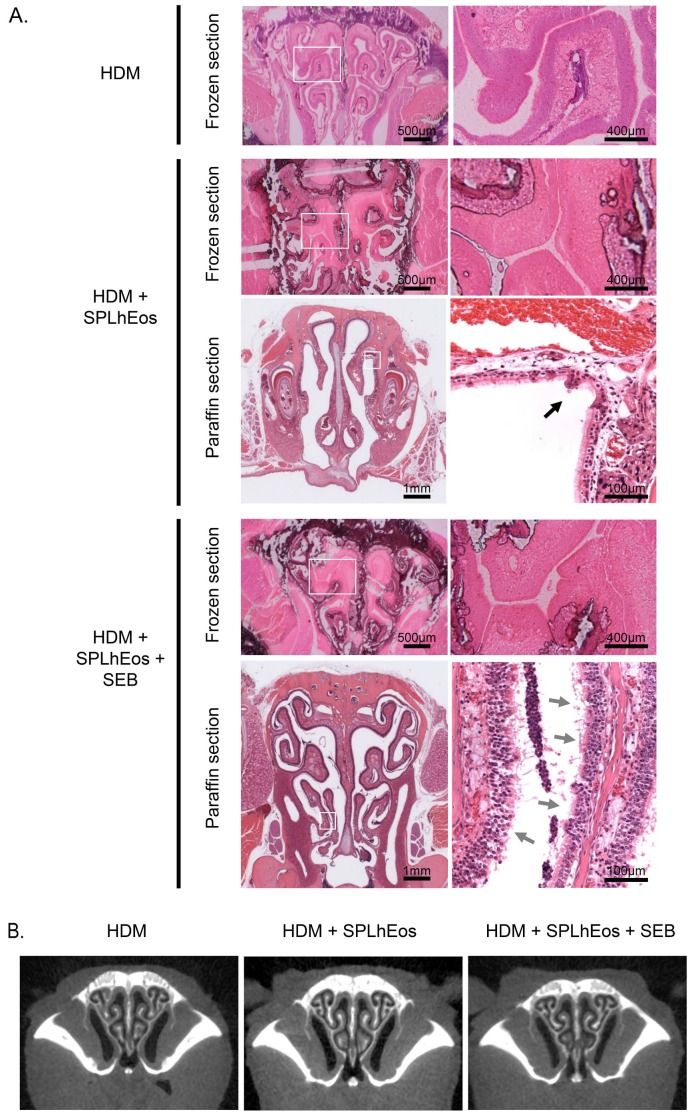
Histological (**A**) and CT (**B**) analyses on the transfer of SPLhEos with house dust mite (HDM). Transfer of HDM alone, HDM with SPLhEos (4 × 10^7^ cells), and HDM with SPLhEos (4 × 10^7^ cells) plus *Staphylococcus aureus* enterotoxin B (SEB) were grouped into the adoptive transfer system (two individual experiments with *n* = 4 or 5 mice per group). Subfigures (**A**,**B**) indicate histology in H&E-stained frozen and paraffin-embedded sections, and coronal images taken by the CT scan in the paranasal sinus, respectively.

As HDMs promote eosinophilic airway inflammation with AHR (Figure S2A) and are implicated in both adaptive and innate immune responses [19,20], donor and recipient mice were sensitized with HDM, and adoptive transfers were performed with HDM. As shown in Figure 3A, upon transfer of SPLhEos activated by HDM and HDM, convex lesions were observed in the epithelial layer of the paranasal sinus with no evidence of nasal polyps present in humans being observed in the paraffin sections of mice also treated with SEB, which modulates innate immunity. Upon transfer of SPLhEos activated by HDM with HDM plus SEB, a more pronounced epithelial injury was observed in paraffin sections. Histological analyses of frozen sections using the unfixed and undecalcified method (reflecting near-physiological morphology) revealed no clear differences in physiological morphology among these groups. Furthermore, swelling of the nasal mucosa was barely observed in any of the groups. Similar results were observed in the CT analysis (Figure 3B).

**Figure 4:**
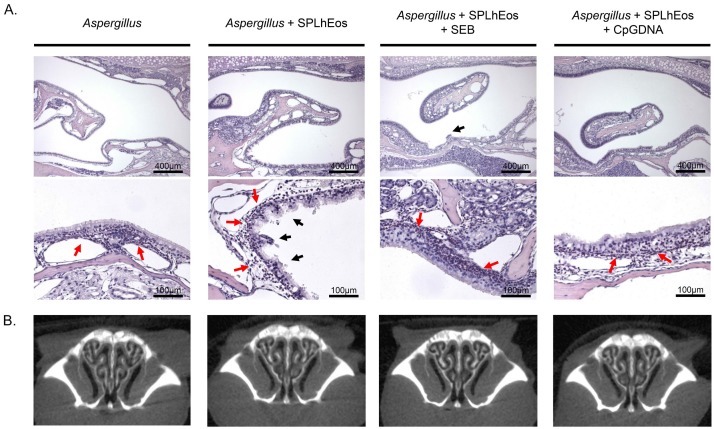
Histological (**A**) and CT (**B**) analyses on the transfer of SPLhEos with *Aspergillus*. Transfer of *Aspergillus* alone, *Aspergillus* with SPLhEos (4 × 10^7^ cells), *Aspergillus* with SPLhEos (4 × 10^7^ cells) plus *Staphylococcus aureus* enterotoxin B (SEB), or *Aspergillus* with SPLhEos (4 × 10^7^ cells) plus DNA containing unmethylated CpG motifs (CpGDNA) were grouped into the adoptive transfer system (*n* = 5 mice per group). Subfigures (**A**,**B**) indicate Sirius-red-stained histological sections and coronal images taken by CT scans in the paranasal sinus, respectively.

Allergic fungal rhinosinusitis, such as *Aspergillus* infection, is an additional subtype in CRSwNP, with the fungus not only promoting eosinophilic airway inflammation with AHR (Figure S2B) similar to OVA and HDM, but also inducing toll-like receptor (TLR) expression [21,22,23]. Therefore, to investigate whether *Aspergillus* induces upper airway inflammation similar to ECRS, adoptive *Aspergillus* with/without SPLhEos was transferred into *Aspergillus*-sensitized recipient mice. As shown in Figure 4, on transfer of *Aspergillus* with SPLhEos, severe epithelial layer injury, numerous convex lesions, and marked eosinophilic infiltration into the mucosal layer were observed compared with *Aspergillus* alone; however, no nasal polyps like in humans were found. To induce a more powerful immune response, SEB or CpGDNA were administered alongside SPLhEos and *Aspergillus* as TLR9 agonists. However, no clear histological differences were observed among SPLhEos with *Aspergillus*, with *Aspergillus* plus SEB, or with *Aspergillus* plus CpGDNA. In micro-CT analysis, nasal polyps, observed in ECRS, were observed in none of the groups. Thus, treatment with SEB or CpGDNA exerted no amplification effects.

### 3.3. Thin Tissue Component of Turbinate in Murine Paranasal Sinuses

**Figure 5:**
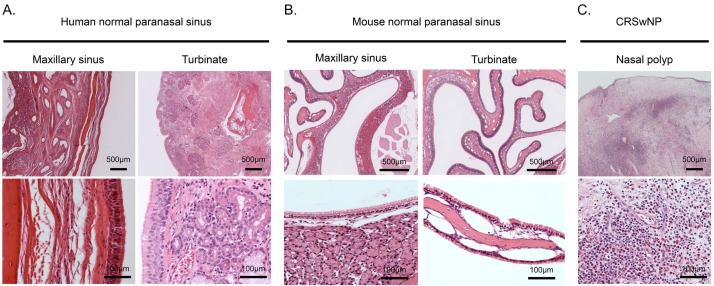
Histology of normal human paranasal sinus (**A**), normal mouse paranasal sinus (**B**), and nasal polyps from chronic rhinosinusitis with nasal polyps (CRSwNP) (**C**). Histological sections were stained using H&E.

Histological differences between human and murine paranasal sinuses are shown in Figure 5. In humans, the turbinate comprises a rich stromal component with serous glands, while the maxillary sinus comprises poor submucosal tissue (Figure 5A). In contrast, the murine tissue composition is the inverse of that in human (Figure 5B); characteristics of histological differences of the paranasal sinus between humans and mice are summarized in Table 1. From histological findings in CRSwNP following ESS, nasal polyps commonly consist of a thin epithelial layer with prominent edema and marked proinflammatory cells (mainly eosinophils) (Figure 5C). However, the nasal polypoid lesions in Figure 3 and Figure 4 seem to comprise epithelial metaplasias with injury of the epithelial layer, rather than nasal polyps in ECRS. 

## 4. Discussion

We aimed to investigate whether eosinophilia during upper airway inflammation induces olfactory disturbance and/or promotes nasal polyp formation in the CRS using an adoptive transfer system with activated SPLhEos. Our results demonstrate that SPLhEos-induced upper airway inflammation results in hyposmia, but morphological alteration of the paranasal sinus was not promoted. This paper is the first to report a direct contribution of eosinophilic upper airway inflammation in olfactory disturbance. Olfactory disturbance is a common symptom of CRSwNP [24], especially as an initial symptom of ECRS [10]. There are two possible mechanisms of olfactory disturbance: (1) closing of the olfactory cleft, which occurs during nasal septum obstruction by nasal polyps, or (2) olfactory neuroepithelium injury following mucosal inflammation [25,26]. Doty et al. reported that there was little correlation between airway patency and olfactory function, except in the case of complete or almost complete blockage of the olfactory cleft, resulting in odorant molecules not gaining access to the olfactory mucosa [27]. Thus, this report suggests that hyposmia in our model is due to mucosal inflammation rather than physical airway obstruction. Our observations of hyposmia without morphological changes or nasal obstruction of the olfactory cleft suggest that hyposmia may be induced by eosinophil-derived toxic mediators following olfactory neuroepithelium injury. This is suggested as eosinophil-derived granules have a capability of inducing tissue damage and dysfunction [1,28]. Causes of hyposmia require further investigation for treatment of patients with ECRS, as no studies have been reported on direct injury of the neuroepithelium by eosinophil-derived mediators.

CRSwNP including ECRS is a heterogeneous disease that is identified by various inflammatory endotypes [8,29,30]; its pathogenesis implicates three major types of innate and adaptive cell-mediated effector immunity: type 1 (natural killer cells, innate lymphoid cell (ILC)1, cytotoxic T (Tc)1, and Th1), type 2 (ILC2, Tc2, and Th2), and type 3 (ILC3, Tc17, and Th17) immune responses [30,31]. Recent reports have suggested that increased expression of TLRs (TLR2, 4, 7, and 9) and protease-activated receptors contributes to the development of CRS [5,32,33]. Furthermore, the expressions of TLR1–7, 9, and 10 have been identified in eosinophils, a key player in CRSwNP [1]. Notably, it has been reported that SEB not only enhances Th2 response through interaction with TLR2 signaling [34,35] but also plays a potential role in IL-5, IL-13, and RANTES production in dispersed nasal polyps following the development of CRSwNP [36]. Supporting this, high concentrations of *S. aureus* enterotoxin-specific IgE are associated with nasal polyps with intense eosinophilic inflammation [8]. Regarding molecular pathogenesis, HDM induces both adaptive and innate immune responses through protease-activated receptor (PAR) 2 via proteases [37], and TLR2 and 4 [19,20], whereas OVA contributes only to antigen-specific reactions, reflecting Th2-adaptive immune responses. Similarly, *Aspergillus* induces the signaling of TLRs (TLR1, 2, 3, 4, 6, and 9) [21,22,23] in a wider range than HDM. Therefore, we also investigated the effects of HDM and *Aspergillus* in the adoptive SPLhEos transfer system. However, no nasal polyps were observed like those seen in patients with CRSwNP, although more severe damage of the epithelial layer was observed following the development of convex lesions.

Moreover, we assess SPLhEos-induced upper airway inflammation because micro-CT analysis can be used to detect the physiological and pathological morphology of the paranasal sinus. Although micro-CT analysis was confirmed as a useful method to detect the swelling of the nasal mucosa following the methacholine challenge test (Figure S3), no morphological changes were observed in this experimental model. Thus, these results indicate that eosinophilic upper airway inflammation in a murine model using an adoptive transfer system does not induce mucosal irregularity with edema like those seen in CT images of patients with CRSwNP.

Regarding a CRSwNP animal model, Kim et al. reported an animal model of CRSwNP through frequent nasal instillation of SEB, CpGDNA, or *Aspergillus* protease under OVA-induced, Th2-skewed immune response, which required >100 days for the development of nasal polyps [12,13,14]. They found not only nasal polyps or polypoid lesions with epithelial thickening and infiltration of inflammatory cells, but also mucosal irregularity of nasal polyps by micro-CT analysis [12,13,14]. We observed convex lesions (polypoid lesion) with hallmark eosinophil infiltration, similar to the findings by Kim et al. However, two different pathologists indicated that these nasal polypoid lesions are similar to reactive granuloma with epithelial hyperplasia, and not the same as nasal polyps in humans. This is because polyps usually consist of edematous mucosa with a loose stroma and a variety of inflammatory cell infiltration (seen in Figure 5C). In this pathological change, a small widened stroma was observed, filled with small/capillary vessels and inflammatory cells, with no edema. Furthermore, as summarized in Table 1, histological structures are completely different between humans and mice. Thus, in a murine model, these findings suggest that it could be experimentally difficult to induce the development of nasal polyps like in humans.

In conclusion, we report that eosinophilic upper airway inflammation induces hyposmia using an adoptive transfer system into the nasal cavity. Analysis of hyposmia using the adoptive SPLhEos transfer model may be a useful examination method for understanding disease mechanisms or developing new drugs for olfactory disorders. Regarding the CRSwNP murine model, our data and previous reports suggest that additional studies are essential, but that caution is required in future investigations when using these models.

## Figures and Tables

**Table 1:** Characteristics of human and mouse paranasal sinuses.

	Maxillary Sinus			Turbinate		
	Mucosal Layer	Interstitium Tissue	Gland	Mucosal Layer	Interstitium Tissue	Gland
Human	Thin	Poor	Poor	Thick	Rich	Rich
Mouse	Thin–moderate	Poor	Rich	Thin	Poor	Poor

## Supplementary Materials

The following are available online at http://www.mdpi.com/2076-3271/7/2/22/s1. Figure S1: Flow cytometric analysis of splenocytes from IL-5 transgenic mouse, Figure S2: Induction of allergic airway inflammation by HDM (A) and Aspergillus (B), Figure S3: CT analysis images in the paranasal sinus. Left and right panels indicate coronal sections in control (PBS/PBS) and OVA-sensitized and challenged groups (OVA/OVA), respectively; and online supplementary methods.

## Abbreviations

AR, allergic rhinitis; Alum, aluminum hydroxide; CRS, chronic rhinosinusitis; CRSwNP, chronic rhinosinusitis with nasal polyp; CRSsNP, chronic rhinosinusitis without nasal polyp; CT, computer tomography; ECP, eosinophil cationic protein; ESS, endoscopic sinus surgery; ECRS, eosinophilic chronic rhinosinusitis; EDN, eosinophil-derived neurotoxin; EPO, eosinophil peroxidase; Der f, *Dermatophagoides farinae*; HDM, house dust mite; i.n., intranasal; i.p., intraperitoneal; i.t., intratracheal; JESREC, Japanese Epidemiological Survey of Refractory Eosinophilic Chronic Rhinosinusitis; ILC, innate lymphoid cell; IL, interleukin; MBP, major basic protein; MCH, methacholine; MMP, matrix metalloproteinase; NK, natural killer; OVA, ovalbumin; PAR, protease-activated receptor; SAE, *Staphylococcus aureus* enterotoxin; SAgs, superantigens; SEA, staphylococcal enterotoxin A; SEB, *Staphylococcus aureus* enterotoxin B; SE-IgE, *Staphylococcus aureus* enterotoxin-specific IgE; SPLhEos, splenocytes including a high number of eosinophils; Tc, cytotoxic T; TGF, transforming growth factor; Tg, transgenic; Th, T helper cell type; TLR, toll-like receptor.
