# Supplementary material for: Eosinophilic Upper Airway Inflammation in a Murine Model Using an Adoptive Transfer System Induces Hyposmia and Epithelial Layer Injury with Convex Lesions
Source: Med Sci (Basel). 2019 Feb 5;7(2):22. doi: 10.3390/medsci7020022 (PMC6409781; doi:10.3390/medsci7020022)
Supplement: Supplementary file 1 [file medsci-07-00022-s001.pdf]

medical sciences

MDPI

Supplementary Materials

Eosinophilic Upper Airway Inflammation in a Murine Model Using an Adoptive Transfer System Induces Hyposmia and Epithelial Layer Injury with Convex Lesions

Akira Kanda [1,2,*,†], Kenji Kondo [3,†], Naoki Hosaka [4], Yoshiki Kobayashi [1,2], Dan Van Bui [1], Yasutaka Yun [1], Kensuke Suzuki [1], Shunsuke Sawada [1], Mikiya Asako [1,2], Akihiko Nakamura [5], Koichi Tomoda [1], Yoshiko Sakata [6], Koji Tsuta [7], David Dombrowicz [8], Hideyuki Kawauchi [9], Shigeharu Fujieda [10] and Hiroshi Iwai [1]

1 Department of Otolaryngology, Head and Neck Surgery, Kansai Medical University, Hirakata, 573-1010, Japan; kobayosh@hirakata.kmu.ac.jp (Y.K.); buivanda@hirakata.kmu.ac.jp (B.V.D.); yunys@hirakata.kmu.ac.jp (Y.Y.); suzukken@hirakata.kmu.ac.jp (K.S.); sawadash@hirakata.kmu.ac.jp (S.S.); asako@hirakata.kmu.ac.jp (M.A.); tomodak@hirakata.kmu.ac.jp (K.T.); iwai@hirakata.kmu.ac.jp (H.I.)
2 Allergy Center, Kansai Medical University, Hirakata, 573-1010, Japan
3 Department of Otolaryngology and Head and Neck Surgery, Graduate School of Medicine, the University of Tokyo Hospital, Tokyo, 113-8655, Japan; kondok-tky@umin.ac.jp
4 Department of Pathology, Fuchu Hospital, Izumi, 594-0076, Japan; hosakan@hirakata.kmu.ac.jp
5 Nakamura ENT Clinic, Sakai, 591-8025, Japan; nakamura-ent@paw.hi-ho.ne.jp
6 Central Research Laboratory, Kansai Medical University, Hirakata, 573-1010, Japan; sakatayo@hirakata.kmu.ac.jp
7 Department of Pathology, Kansai Medical University, Hirakata, 573-1010, Japan; tsutakoj@hirakata.kmu.ac.jp
8 EGID, Inserm, CHU Lille, Institut Pasteur de Lille, U1011, University of Lille, Lille 59019, France; david.dombrowicz@pasteur-lille.fr
9 Department of Otorhinolaryngology, Shimane University Faculty of Medicine, Izumo, 693-0021, Japan; kawauchi@med.shimane-u.ac.jp
10 Department of Otorhinolaryngology Head & Neck Surgery, University of Fukui, Fukui 910-1193, Japan; sfujieda@g.u-fukui.ac.jp
* Correspondence: akanda@hirakata.kmu.ac.jp; Tel.: +81-72-804-0101
† Equally contributed authors.

1. Online supplementary methods

Flow cytometry

Staining were performed with Siglec-F and Gr-1 (BD Biosciences, New Jersey, USA) for 30 minutes at 4°C on 5x10^5 cells in 100μL after blocking nonspecific binding to FcγR with anti-CD16/CD32 (2.4G2). Stained cells were separated and counted using flow cytometry (BD FACSCanto™ II) and FlowJo software (Tomy Digital Biology, Tokyo, Japan).

Induction of allergic rhinitis with asthma

Mice (BALB/c) were sensitized through two intraperitoneal (i.p.) injections of PBS, 50 μg OVA (grade V; Sigma), 10 μg Der f (Institute of Tokyo Environmental Allergy) as HDM antigen, or 50 μg *Aspergillus* (Institute of Tokyo Environmental Allergy) in 2 mg of aluminum hydroxide (Thermo

Fisher Scientific) at days 0 and 14. Mice were challenged 4 times corresponding to their sensitization, with 30 μL PBS, 0.5% OVA, 1 μg HDM, or 10 μg *Aspergillus* via i.n. administration into both nostrils on days 21–24. Administration was performed under systemic anesthesia with 0.5 mg/kg medetomidine (Domitor; Pfizer) and 50 mg/kg ketamine (Ketalar; Daiichi-Sankyo). Functional assays of asthma, such as AHR measurement and bronchoalveolar lavage fluid (BALF), and CT analysis in paranasal sinus were performed.

AHR and BAL analysis in model of allergic rhinitis with asthma

Measurement of resistance and compliance reflecting AHR were preformed according to our previous our report [1]. To count the infiltrating cell numbers and analyze the populations of cell types (eosinophils, neutrophils, macrophages, and lymphocytes), bronchoalveolar lavage fluid (BALF) analysis was performed.

CT analysis in paranasal sinus in model of allergic rhinitis with asthma

To measure the swelling of nasal mucosa in the paranasal sinus of naive tissue or the model of AR with asthma, CT scans were performed immediately using a Siemens Inveon Micro-CT 15 min after treatment with methacholine (2 mg/ml) or PBS on day 26. Cross-sectional images were then reconstructed using Inveon Viewer QuickLaunch software and converted to DICOM format with PMOD software.

Statistical analyses

Data are presented as the means ± SEMs. Statistical significance was determined using the Mann–Whitney U test, except for invasive plethysmographic data, which were assessed using repeated-measures ANOVA.

2. Online supplementary figure legends

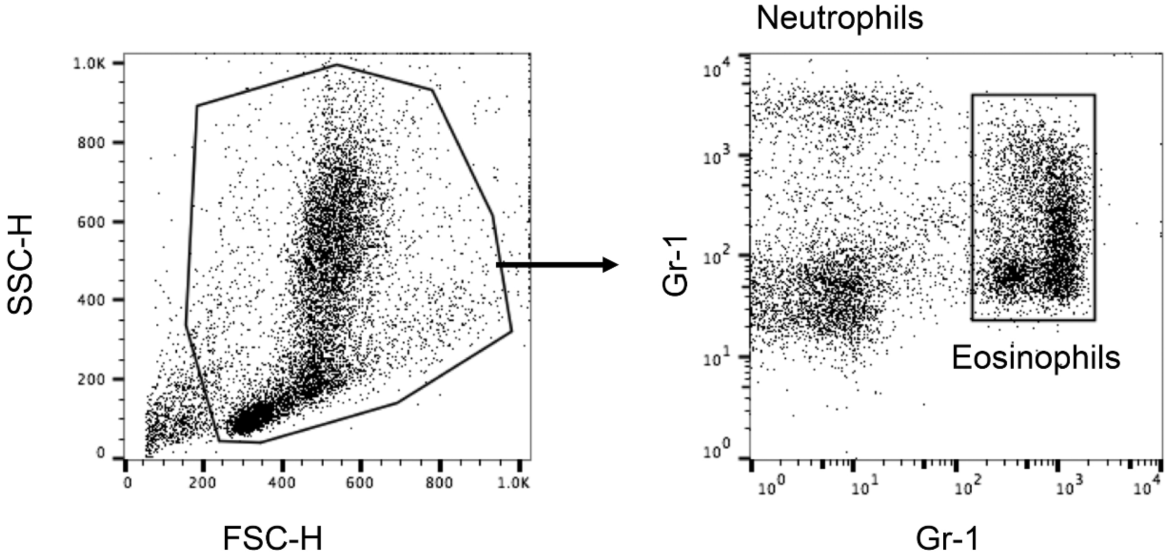

Figure S1. Flow cytometric analysis of splenocytes from IL-5 transgenic mouse. Eosinophils and neutrophils were identified as Siglec-F^{higih} Gr-1^{middle} and Siglec-F^{-} Gr-1^{high}, respectively.

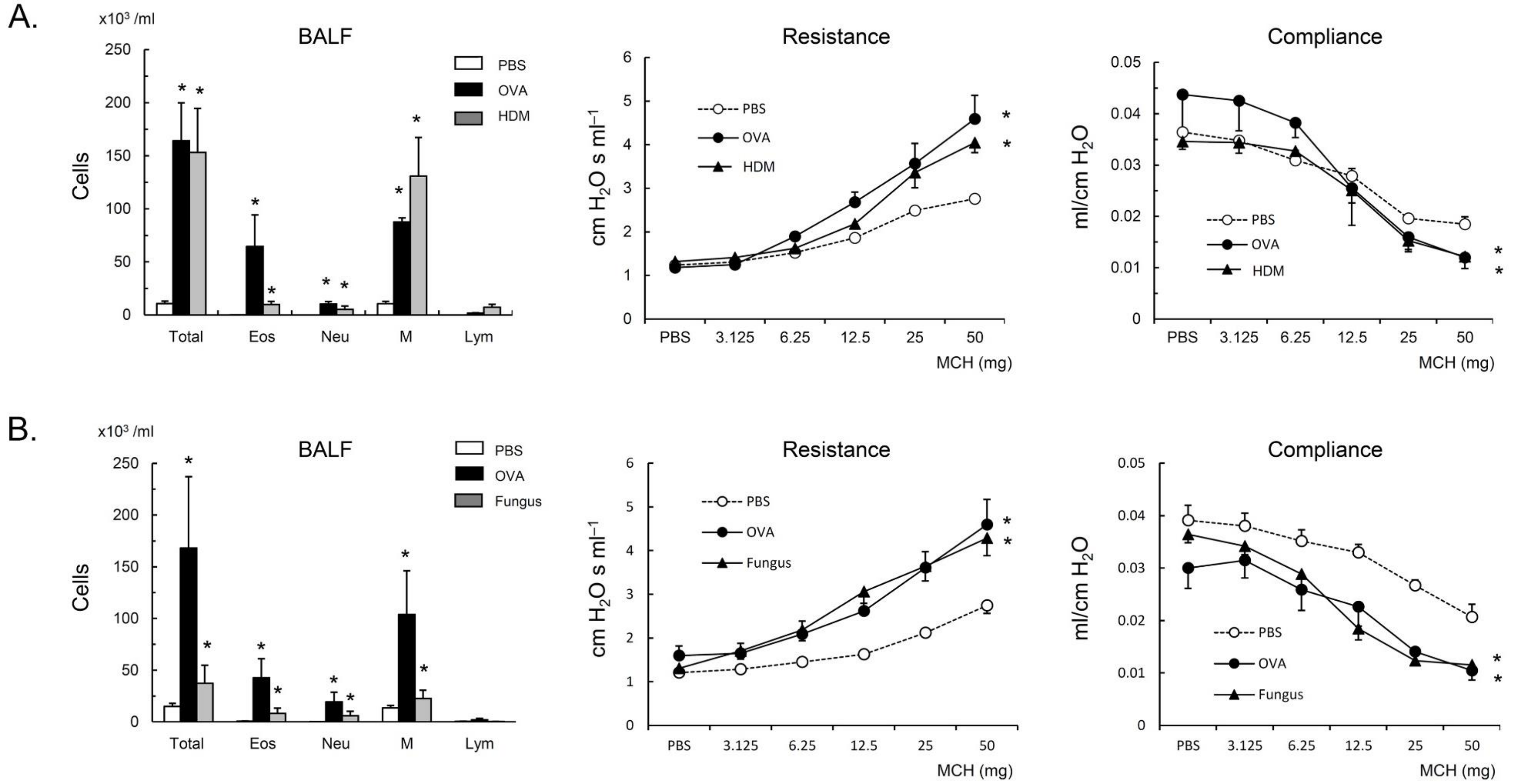

Figure S2. Induction of allergic airway inflammation by HDM (**A**) and *Aspergillus* (**B**). Bronchoalveolar lavage fluid (BALF) cellularity: Total cells, eosinophils (Eos), neutrophils (Neu), macrophages (M), and lymphocytes (Lym). Methacholine-induced airway hyperresponsiveness (AHR) in the lower airway was shown as assessed by measurements of airway resistance and compliance. Data (n = 4–5) are expressed as the means ± SEM. *Statistically significant differences from control mice ($P < 0.05$).

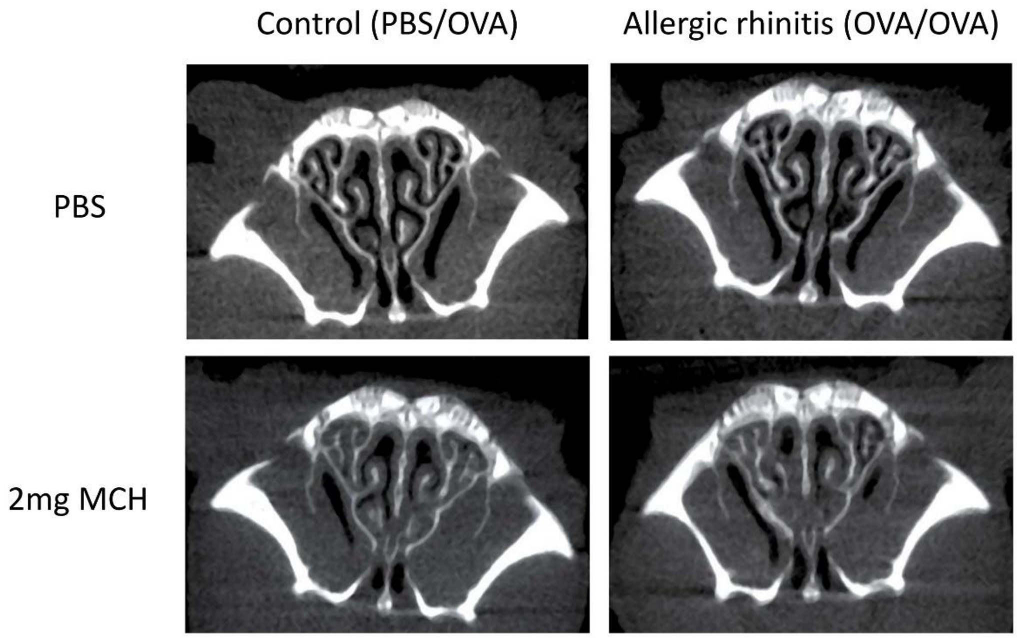

Figure S3. CT analysis images in the paranasal sinus. Left and right panels indicate coronal sections in control (PBS/PBS) and OVA-sensitized and challenged groups (OVA/OVA), respectively. Upper and lower panels show treatment with 30 μL PBS and 2 mg/ml methacholine (MCH) into the nasal cavity, respectively. Gray and white levels in the CT image of the paranasal sinus indicate the nasal mucosa and bone, respectively.
